# Gastroduodenal intussusception as a rare complication of gastrointestinal stromal tumor: a case report

**DOI:** 10.11604/pamj.2024.48.143.43409

**Published:** 2024-08-01

**Authors:** Mujaheed Suleman, Jay Lodhia, Bahati Robert Kitandu, Ayesiga Herman

**Affiliations:** 1 Department of General Surgery, Kilimanjaro Christian Medical Centre, Box 3010, Moshi, Tanzania,; 2 Faculty of Medicine, Kilimanjaro Christian Medical University College, Box 2240, Moshi, Tanzania

**Keywords:** Gastroduodenal intussusception, gastrointestinal stromal tumor, lead point, intussusception, case report

## Abstract

Intussusception is a common and well-known surgical pathology. Intussusception in adults is rare, accounting for only 5% of all intussusceptions. Gastrointestinal stromal tumors (GIST) are common mesenchymal tumors of the stomach that can act as a pathological lead point for intussusception. Herein, we present an uncommon case of gastroduodenal intussusception in an elderly female patient due to gastric GIST which was successfully managed surgically by partial gastric resection. This case report creates awareness that clinicians should have gastric intussusception as their differential diagnosis for gastric outlet obstruct and highlights the importance of further radiological investigations.

## Introduction

Intussusception refers to the telescoping of a portion of the gut into the lumen of the immediate adjacent bowel leading to an obstruction in most cases [1]. As commonly seen in the pediatric population, intussusception in adults is rare with identifiable etiologies compared to idiopathic causes in pediatrics. The most common sites in adults involve the small bowel, followed by the large bowel, and rarely the stomach [2]. Gastroduodenal intussusception also known as gastroduodenal invagination or ball valve syndrome, refers to a prolapse of the stomach through the pylorus and into the duodenum, with most cases presenting as gastric outlet obstruction [3,4]. Herein we report a rare case of gastroduodenal intussusception with a pathologic lead point being a GIST in the fundus of the stomach.

## Patient and observation

**Patient information:** a 75-year-old female attended our outpatient clinic presenting with a 1-year history of intermittent abdominal pain. She is a known patient with diabetes on regular oral medications. The pain was episodic, more localized in the epigastric region radiating to the umbilicus, associated with emesis of recently eaten food particles and saliva, and reported of significant weight loss of 10 kgs.

**Clinical findings:** on examination, she was wasted, mildly pale, not dyspneic, not jaundiced, moderately dehydrated, with vitals within normal range and an axillary temperature of 36°C, and was saturating at 99% on room air. Her abdomen was flat with symmetrical contours, and an epigastric mass was appreciated on palpation which was non-tender, 6 by 7 cm, smooth, firm, and mobile.

**Timeline of current episode:** the patient´s index symptoms were for one year, intermittent in nature.

**Figure 1:**
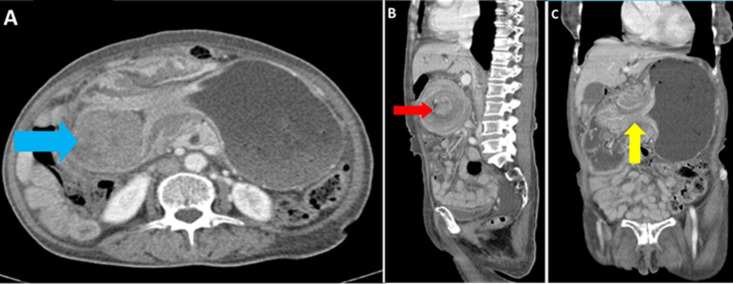
A) CT-scan axial view showing distended stomach with a gastric mass (pathological lead point) (blue arrow); B) saggital view showing ‘target sign’ (red arrow); C) coronal view showing gastroduodenal intussusception (yellow arrow)

**Diagnostic assessment:** her laboratory investigations revealed mild anemia of 10.7 g/dl, normal serum creatinine of 80 µmol/L, and normal levels of serum potassium, sodium, chloride, and liver enzymes. Her abdominal-pelvic CT-scan revealed a distended stomach with an intramural mass arising from the first part of the duodenum measuring 5.84x5.55x5.67 cm in size, causing the gastric outlet obstruction suggestive of an intussusception vs duodenal mass (Figure 1).

**Diagnosis:** presumptive diagnosis was gastric outlet obstruction secondary to gastric intussusception due to gastrointestinal stromal tumor from histology, with good prognosis it was localized in the stomach and wholly resected.

**Figure 2:**
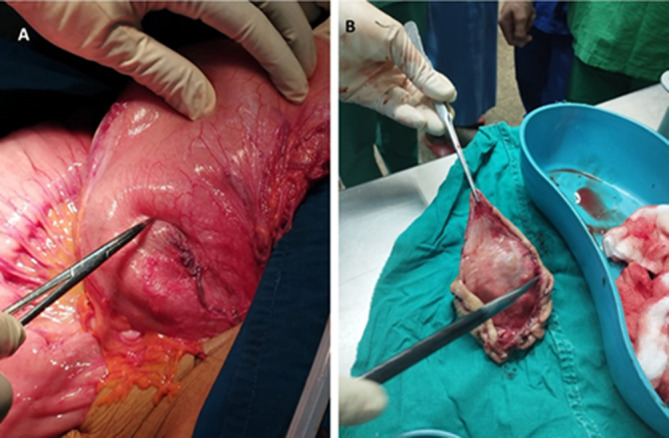
A) photograph showing gastroduodenal intussusception (probe); B) gastric mass (GIST) as pathological lead point

**Therapeutic intervention:** after thorough counseling, she was then taken for laparotomy. Intra-operatively a gastro-duodenal intussusception was appreciated with a 6 by 6 cm firm gastric mass arising from the fundus of the stomach (pathological lead point) (Figure 2). There were also approximately 1000 mls of clear amber-colored ascites. Her liver and spleen were grossly normal looking. The hepatic flexure of the colon was kocherised and freed and the lesser sac was opened. The gastric intussusception was successfully reduced manually. A wedge resection of the gastric mass using a GI stapler was performed and the edges were over sewn using absorbable sutures, hemostasis was achieved and the abdomen was closed in layers.

**Figure 3:**
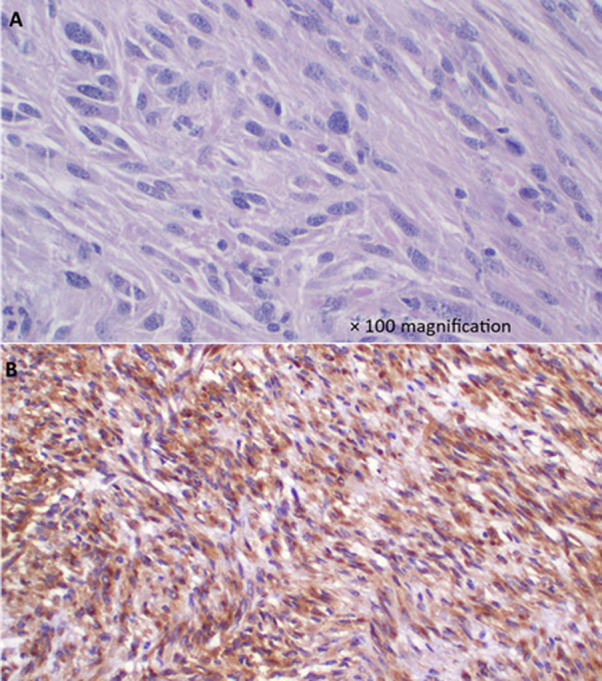
A) GIST highlighting mild to moderate pleomorphic oval or spindle-shaped cells with abundant eosinophilic cytoplasm and occasional mitoses (H&E staining); B) GIST demonstrating CD117 positive diffuse cytoplasmic staining (IHC, x63)

**Follow-up and outcomes:** postoperatively, she was nursed in the general ward with good recovery and was discharged on day 5 with no gastrointestinal symptoms. She was then reviewed three weeks later at the outpatient unit where she was faring well and the incision was healed. Histology results of the resected mass revealed a GIST (Figure 3) therefore she was then referred to the oncologists for continuum of management.

**Patient perspective:** on reviewing the patient at the surgical outpatient unit, the patient was rather pleased with gratitude towards the care and counseling provided by the attending clinicians.

**Informed consent:** written informed consent was obtained from the patient for publication in this case report; additionally, accompanying images have been censored to ensure that the patient cannot be identified. A copy of the consent is available on record.

## Discussion

The earliest known description of intussusception was made by the Dutch physician Paul Barbette in 1674, with successful manual reduction of an intussusception being first performed by Sir Jonathan Hutchinson in 1871 in a 2-year-old girl following failure of hydrostatic reduction [5,6]. Intussusception in adults is rare, accounting for only 10% of all cases, and 5% being gastric, 90% have a secondary lead point while only 10% are idiopathic [1,4]. Several lead points have been described in literature varying from benign to malignant causes, including GISTS, gastric polyps, gastric lipomas, and gastric carcinomas [7].

Gastrointestinal stromal tumors are the most common cause of gastroduodenal intussusception, with less than 30 reported cases. They present with varying degrees of obstruction; hence, give a diagnostic dilemma due to their vague subclinical and chronic presentation including intermittent, mild, to severe epigastric pain, nausea, vomiting, abdominal tenderness, palpable epigastric mass, weight loss plus gastrointestinal bleeding with anemia secondary to mucosal ulceration, and in some cases complicating to acute pancreatitis [2,3,7,8]. This was evident in our case whereby the patient suffered for 1 year with unspecific abdominal symptoms.

Gastrointestinal stromal tumors causing gastroduodenal intussusception are predominantly seen in females (76.5%) and include a median age of 65 years [8]. Gastrointestinal stromal tumors are mesenchymal tumors of the gastrointestinal tract and are pathologically defined by positive immunostaining for c-kit proto-oncogene - CD117 (overexpressed in 95%) and CD34 (positive in 60% to 70%) [9].

Various diagnostic modalities are capable for the diagnosis of GIST and other lesions causing the intussusception including CT-scan, MRI, and upper gastrointestinal endoscopy. Features of intussusception in a CT-scan include a target sign or a sausage-shaped mass in the longitudinal axis, and can also aid in staging the disease [7]. A target sign was seen in our case and aided in ruling out the presence of metastasis.

The mainstay of treatment is surgical in most cases, aiming at R_0_ resection with options including laparotomy, laparoscopy, and endoscopy as reported first in 2017 in Japan (ESD) [6,8,10]. Mitotic index and tumor size are the main variables used in risk stratification as introduced by the National Institute of Health (NIH), also called Fletcher´s criteria [9]. Our patient belonged to the intermediate risk group according to the NIH-Fletcher´s criteria. The tyrosine kinase inhibitor imatinib mesylate is used in the management of GISTs and has been shown to reduce recurrence when used as an adjuvant to surgical resection (6% recurrence on imatinib vs. 17% without therapy at 12 months). Prognosis is better with GISTs compared to other gastric tumors with a 5-year survival rate of 48-70% [7].

## Conclusion

A gastroduodenal intussusception is a rare form of adult intussusception with a variety of lead points, GISTs being the highest on the list. Gastroduodenal intussusception causes varying degrees of clinical symptoms causing a diagnostic dilemma. Due to the rarity of this condition, a high index of suspicion can aid in early diagnosis and care and reduce morbidity and mortality due to complications that ensue.
